# Prevalence of anemia and deficiency of iron, folic acid, and zinc in children younger than 2 years of age who use the health services provided by the Mexican Social Security Institute

**DOI:** 10.1186/1471-2458-7-345

**Published:** 2007-11-30

**Authors:** Ximena Duque, Sergio Flores-Hernández, Samuel Flores-Huerta, Ignacio Méndez-Ramírez, Sergio Muñoz, Bernardo Turnbull, Gloria Martínez-Andrade, Rosa I Ramos, Marco González-Unzaga, María E Mendoza, Homero Martínez

**Affiliations:** 1 Unidad de Investigación en Epidemiología Nutricional, Instituto Mexicano del Seguro Social, Mexico D.F., Mexico; 2 Departamento de Salud Comunitaria, Hospital Infantil de México "Federico Gómez", Mexico D.F., Mexico; 3 Instituto de Investigaciones en Matemáticas Aplicadas y en Sistemas, Universidad Nacional Autónoma de México, Mexico D.F., Mexico; 4 Facultad de Medicina, Universidad de la Frontera, Temuco, Chile; 5 RAND, Santa Monica, CA, USA; 6 Dirección de Investigación Médica, Hospital Infantil de México "Federico Gómez", Mexico D.F., Mexico

## Abstract

**Background:**

In Mexico, as in other developing countries, micronutrient deficiencies are common in infants between 6 and 24 months of age and are an important public health problem. The objective of this study was to determine the prevalence of anemia and of iron, folic acid, and zinc deficiencies in Mexican children under 2 years of age who use the health care services provided by the Mexican Institute for Social Security (IMSS).

**Methods:**

A nationwide survey was conducted with a representative sample of children younger than 2 years of age, beneficiaries, and users of health care services provided by IMSS through its regular regimen (located in urban populations) and its Oportunidades program (services offered in rural areas). A subsample of 4,955 clinically healthy children was studied to determine their micronutrient status. A venous blood sample was drawn to determine hemoglobin, serum ferritin, percent of transferrin saturation, zinc, and folic acid. Descriptive statistics include point estimates and 95% confidence intervals for the sample and projections for the larger population from which the sample was drawn.

**Results:**

Twenty percent of children younger than 2 years of age had anemia, and 27.8% (rural) to 32.6% (urban) had iron deficiency; more than 50% of anemia was not associated with low ferritin concentrations. Iron stores were more depleted as age increased. Low serum zinc and folic acid deficiencies were 28% and 10%, respectively, in the urban areas, and 13% and 8%, respectively, in rural areas. The prevalence of simultaneous iron and zinc deficiencies was 9.2% and 2.7% in urban and rural areas. Children with anemia have higher percentages of folic acid deficiency than children with normal iron status.

**Conclusion:**

Iron and zinc deficiencies constitute the principal micronutrient deficiencies in Mexican children younger than 2 years old who use the health care services provided by IMSS. Anemia not associated with low ferritin values was more prevalent than iron-deficiency anemia. The presence of micronutrient deficiencies at this early age calls for effective preventive public nutrition programs to address them.

## Background

The first 2 years of life are crucial for children's present and future health and nutritional status and, more specifically, for their mental, physical, and emotional development. In the last decade, micronutrient deficiencies have received much attention as it has been demonstrated that even subclinical states, such as mild iron deficiency or low concentrations of zinc, are associated with functional outcomes [1]. These include, among others, impaired psychomotor development [2,3], decreased work capacity, diminished immunological response, and linear growth retardation [4-6]. Likewise, mild zinc deficiency has a negative influence on growth and development and increases the risk of diarrhea and acute respiratory infections [5,7-9]. Folic acid deficiency affects erythropoiesis, which, in turn, may be responsible for macrocytic anemia [10].

In Mexico, as in other developing countries, micronutrient deficiencies occur mainly between 6 and 24 months of age and are a significant public health problem [11-15]. Children under 2 have increased nutritional requirements because of their growth spurt, which often leads to a negative nutrient balance [16,17]. Preterm babies, as well as those born small for gestational age, are particularly vulnerable to iron deficiency in their first months [17]. During the first year of life, another risk factor for nutritional deficiencies occurs from inadequate complementary feeding practices. These are characterized by consumption of foods with low amounts of bioavailable micronutrients as well as with inhibitors of their absorption, and the practices often extend up to the age of 2 years. In the cases of iron and zinc, risk of inadequate intake is increased in countries where diets at this age include cereals with a high content of phytic acid coupled with low intake of animal foods [1,7].

According to results from the National Nutrition Survey (NNS) in Mexico, carried out in 1999 (NNS-99), the highest prevalence of anemia was found in children between the ages of 12 and 24 months (48.9%); there was a slightly higher prevalence in rural areas (52.9%) as compared to urban ones (46.8%) [15]. In children under 5 years old, 35% of anemia cases were not associated with iron deficiency but rather with one or more vitamin deficiencies [13]. In children between 1 and 2, 66.6% of children had iron deficiency, as estimated by the percentage of transferrin saturation [13]. Folic acid deficiency was estimated in 8.8% of children under 2 and zinc deficiency in 34% [13,14]. However, NNS-99 was not designed to be representative of children under 2 years old, so results for this age group are based on very small sample sizes.

When the study was conducted, the population of Mexico was approximately 100 million people. More than half of them were right-holders of the Instituto Mexicano del Seguro Social-IMSS (Mexican Institute for Social Security). Through two branches, IMSS provides social, welfare, and medical benefits for nongovernment employees and their direct dependents as well as for the underserved population living in rural or semi-rural areas. The regular regimen (RR) provides care to salaried employees, whereas IMSS-Oportunidades (IO) provides care to those in underserved, rural/semi-rural areas.

The present survey was carried out in primary health care-level facilities, which offer services for infants and children such as medical care for common childhood illnesses (i.e., the common cold, acute diarrhea) as well as well-baby clinic, including growth monitoring, promotion and support of breast-feeding, and primary preventive actions (i.e., vaccination). Additionally, several government-sponsored programs geared to the underserved population are offered through IO program like distribution of fortified foods for pregnant women, infants, and malnourished children. In communities where IO operated, the whole community is a beneficiary of this program and no other public medical services are available, making it unlikely that people living in those communities will use other services. Also, IO has a strong component of home visits and community development activities, which tend to strengthen the relationship between health providers and community members.

This study was commissioned by the IMSS to learn about the prevalence of anemia and micronutrient deficiencies, particularly of iron, folic acid, and zinc, in children under 2 years of age; it was part of a larger study to document the general health and nutritional status of this age group. To this end, we carried out a national survey, drawing two representative samples of children under 2 years of age, beneficiaries of RR and IO. For each of these populations, the sampling frame was designed to be representative of the four regions in which the country has been divided by other national surveys, which take into consideration differences in socioeconomic development (i.e., North, Center, South, and Mexico City (the nation's capital) with its surrounding periurban area, which includes the Federal District and some municipalities of the surrounding State of Mexico); IO does not operate in this last region, as no major semi-rural or indigenous population lives there. Within each of these regions, RR is based in urban areas, while IO provides its services in the rural/semi-rural areas. We expect that the results of this survey will provide the basis for public health interventions and program strengthening, oriented to alleviate and prevent micronutrient deficiencies in the target population.

## Methods

The survey was carried out between 1999 and 2001 [18]. From this larger study, which included 35,997 children younger than 24 months of age, a subsample was selected to determine micronutrient status to assess the prevalence of iron, folic acid, zinc deficiency, and anemia. Regarding ethical considerations, the Institutional Research Review Board approved the protocol study and, the informed consent letter was signed by mothers of the children in the subsample. The larger sample followed a stratified, two-staged random model by which we obtained a representative sample for each of the two care regimens as well as for each of the four regions of the country that have been used by other national surveys in the past. The sampling frame from which primary sampling units were selected was drawn from a list of all primary health care units in the country, which included 1,160 family medicine units (FMU) for RR and 3,367 rural medical units (RMU) for IO. FMUs were organized by region and number of attending physicians in each of them; RMUs were organized by region and number of children under 2 living in the communities where these units were located. Secondary sampling units were drawn from the children younger than 2 years old who attended the out-patient clinic, vaccination clinic, and well-baby clinic, all based in the primary health care units already described.

### Stratification of primary sampling units

In RR, we defined five strata based on the number of physicians in each FMU. In each region, the sample size for each stratum was proportionally distributed by the number of children under 2 years of age, according to the 1997 census. In IO, we defined three strata, according to the number of children under 2 who lived in communities where the RMUs were located. In each region, sample size in each stratum was proportionally distributed by the number of children younger than 2 registered in 1998. In each stratum, we randomly selected one or more primary sampling units to complete the required sample size.

The sample size for the whole study was calculated based on the estimate of known prevalence in a finite population, according to the prevalence of moderate and severe malnutrition based on weight-for-height criteria, which was 5.3%, according to the NNS-1988 [19]. The subsample required to determine the prevalence of anemia and the deficiencies of iron, zinc, and folic acid was also calculated with a formula to estimate population prevalence under the following assumptions: confidence level 95%, maximum allowable error between the estimate and the parameter: 5%; the resulting sample size was multiplied by a factor of 2.0 because of study design; a 35% overestimate was calculated to account for potential hemolysis of the biological samples as well as for nonrespondents.

The only published information available at the time of the study related to micronutrient deficiency at country level was the prevalence of anemia found in NNS-99, which showed 48% prevalence for children between the ages of 12 and 23 months [20]; although not exactly on the same age range, this was the only available information to use. The resulting sample size was 1,030 children for each region. The sample size was weighted by the proportion with which each primary sampling unit contributed to the overall sample. The overall sample for RR was 4,120 children, and for IO, 3,090 children because an IO is not included in the Mexico City region.

### Data collection

Data for the present study were collected between May 2000 and November 2001. In a series of structured interviews, we collected information on socioeconomic status; birth weight, size, and gestational age; complementary feeding; immunization status; morbidity on the month preceding the interview; nutritional anthropometric measures; and psychomotor development. Preliminary information has been published elsewhere [18]. Only parents who reported no infectious disease in their children in the two-week period preceding the interview were invited to participate in the micronutrient study. Those who agreed were asked to sign informed consents.

### Biological samples

Three ml of venous blood were collected by droplets obtained from venipuncture by trained personnel. They were collected in 250–500 micro liter tubes filled with EDTA K2. Hemoglobin was determined by cyanide-metahemoglobin. Three amber-colored 800 micro-liter trace-element-free micro-tubes with separating gel were used to collect samples for determination of: ferritin by a two-tier immunoradiometric assay (CTK-IRMA, DiaSorin)–percentage of transferrin saturation and zinc by atomic mass spectrometry (AAnalyst 300, Perkin-Elmer, EUA). Four blood droplets were collected in filter paper to determine folic acid by a microbiological method using *Lactobacillus casei *[21]. Red blood cell analyses were carried out in each of the medical care unit laboratories. The blood samples obtained in the amber-colored micro-tubes were centrifuged for 20 minutes at 3000 rpm and serum was separated. Samples for folic acid determination were saved in filter paper (Schleicher & Schuell, No. 903), protected from light, and kept in dark envelopes with desiccant in plastic bags. Samples were kept frozen at -20°C and sent for analysis at a Central facility where they were kept at -70°C until analyzed. All tests were run with the quality control samples recommended by the manufacturer. The standards set by the National Institute of Standards and Technology were used for the Fe, Zn, and folic acid analyses: the coefficient of variation for serum Fe was <5%, for Zn < 10%, and for folic acid <15%. Determinations of ferritin, Fe, and Zn were run against known values from control serum obtained from a pool of healthy donors with coefficients of variation <10%.

#### Cut-offs

Anemia: Hemoglobin values were adjusted for altitude with the following formula: %Hb = [93.3197 (10 ^(0.0000251)(altitude)^)] [22]. Cut-offs for hemoglobin (Hb) concentration to classify anemia were: Hb < 13.5 g/dL for children under 2 weeks of age; Hb< 12.5 g/dL for children between 2 and 3 weeks; Hb < 10 g/dL for children between 1 month and < than 2 months; Hb <9 g/dL between 2 and < 3 months; Hb < 9.5 between 3 and < 6 months; Hb<10.5 g/dL between 6 and < 12 months; and Hb <10.7 g/dL for children between 12 and 23 months [23,24].

Iron deficiency: A cut-off of <10 μg/L of ferritin was used [25].

Folic acid deficiency: Folic acid concentration = 57 ng/mL in whole blood [14].

Low zinc concentration: Serum zinc <65 μg/dL [13,26].

### Statistical analyses

Estimates of the prevalence of anemia, as well as deficiencies in iron, folic acid, and zinc, were carried out for the national and regional levels by age group, taking into consideration the sampling design. In other words, inferences for the whole population considered the stratified two-stage design to apply expansion factors to the sampled population. Each point estimate is presented with 95% confidence intervals (CI). Statistical comparisons between regions and care regimens was carried out with Pearson's chi-square statistics, using the analyses routines for complex surveys; differences between means were assessed using linear models within the same routine to take into consideration the sampling design [27,28]. All statistical analyses were carried out with STATA version 8, special edition [29].

## Results

Response rates were 64.2% (*n *= 2,646) in RR and 74.7% (*n *= 2,309) in IO. Response rates differed in different regions of the country. For RR in the North region, we were able to recruit 76.2% of the expected sample; in the Center, 73.9%; in the South, 58.7%; and in Mexico City and periurban area, 44.8%. For IO, the North recruited 85.8% of the expected sample; the Center, 93.6%; and the South, 44.8%. Children who participated in the micronutrient sample were significantly older than children who participated in the other parts of the larger study. In IO, there was also a significant difference in weight for age: children participating in the micronutrient sample had lower Z score (-0.604) than those in the larger sample (-0.318). The other characteristics–including sex, height for age, years of education of either parent, and percent of working mothers' or fathers' occupation–showed no statistically significant differences between the micronutrient and the general sample (Table 1). Between regions, the only significant difference was found in the years of schooling of RR of mothers coming from the North, which were less than in the rest of the sample.

**Table 1:** General characteristics of children studied for micronutrient deficiencies and children participating in the total survey

	**Regular Regimen (RR)**				**IMSS-Oportunidades (IO)**			
	**Micronutrient sample**		**Survey sample**		**Micronutrient sample**		**Survey sample**	

	Mean	95% CI	Mean	95% CI	Mean	95% CI	Mean	95% CI

Age (months)	11.4	10.6, 13.4	8.6^a^	8.0, 9.2	13.4	12.3, 14.6	10.7^a^	12.3, 14.6
< 6 (%)	13.2	8.5, 19.7	37.7	33.0, 42.7	4.9	2.8, 8.2	27.6	24.6, 30.8
6–11	38.2	34.8, 41.7	38.2	30.6, 34.6	31.0	24.0, 39.1	27.1	25.6, 28.7
12–23	48.7	41.8, 55.6	29.7	26.1, 33.6	64.1	57.4, 70.2	45.3	42.4, 48.3
Sex Male (%)	50.4	48.4, 52.4	51.5	50.6, 52.4	51.8	48.8, 54.8	51.2	49.5, 52.9
Weight for age (Z score)	-0.151	-0.308, 0.005	0.072	-0.04, 0.188	-0.604	-0.615, -0.393	-0.318^b^	-0.454, -0.186
Height for age (Z score)	-0.188	-0.297, -0.078	-0.119	-0.278, 0.039	-1.164	-1.585, -0.743	-0.873	-1.052, -0.694
**Characteristics of mothers**								
Years of study	9.0	8.5, 9.6	9.2	8.8, 9.5	5.3	4.4, 6.1	5.3	4.8, 5.7
Work outside home (%)	24.4	18.4, 31.5	25.6	21.8, 29.8	8.2	6.5, 10.3	9.8	7.2, 13.2
**Characteristics of fathers**								
Years of study	9.2	8.7, 9.7	9.3	8.9, 9.7	5.3	4.5, 6.2	5.5	5.1, 5.9
Occupation (%):								
General services	3.6	3.1, 5.2	4.0	2.3, 5.6	1.3	0.9, 2.0	1.2	0.9, 1.6
Manual laborer	27.4	23.2, 28.4	25.7	23.1, 32.1	29.0	21.9, 37.2	28.6	21.2, 37.4
Clerk	51.4	48.8, 58.0	53.4	43.0, 59.8	7.8	4.7, 12.6	7.3	4.6, 11.6
Small business owner	4.6	4.2, 5.8	4.9	3.2, 4.2	4.4	2.8, 6.9	4.6	2.8, 7.5
Technician	6.7	4.7, 6.4	5.5	5.3, 8.3	0.7	0.4, 1.1	0.6	0.3, 1.0
Professional	5.2	3.7, 5.6	4.5	3.0, 9.0	2.1	1.4, 3.1	2.2	1.4, 3.4
Art and culture	0.4	0.3, 0.7	0.5	0.2, 1.0	0.3	0.2, 0.5	0.3	0.1, 0.5
Land and agriculture	0.7	0.8, 2.5	1.4	0.3, 1.6	52.9	43.6, 62.0	53.7	44.8, 62.4
Migrant					1.3	0.7, 2.6	1.5	0.7, 2.9

95% CI: 95% confidence interval

^a^*p *< 0.001 ^b^*p *= 0.018

At the time of this survey, 84% of children in RR and 94% of children in IO had attended the health care facility searching for some kind of preventive service, like a well-baby clinic or routine vaccination. Only 15.7% of children in RR and 6.4% in IO attended because of an acute illness. Additionally, 20% of mothers reported that the child had had one or more signs of acute infection (e.g., diarrhea, cough, nasal discharge, fever) during the two weeks before the study, which was an exclusion criterion for the micronutrient sample, as acute infections affect the serum status of zinc and iron, making interpretation difficult.

There were some sociodemographic differences in the study sample when comparing regimen by region. Parents' schooling was higher in RR as compared with IO; a larger percentage of mothers in RR worked outside home (24.4% *vs*. 8.2%); and the proportion of houses with dirt floors was lower in RR than in IO (4.3% *vs*. 33.1%). Other differences included: at 4 months of age, 23.8% of children in RR were exclusively breast-fed, in comparison with 46.2% in IO; at this age, children in the South had a higher prevalence of exclusive breast feeding compared to the rest of the country (31.5% in RR and 48.4% in IO); the proportion of children still receiving some breast milk at 12 months of age was larger in the South than in the rest of the country: 26.9% in RR and 66.2% in IO. Lastly, the prevalence of growth stunting in the country was 6.5% in RR, in contrast with 22.4% in IO, and growth stunting was more prevalent in the North for RR (7.4%) and in the Center for IO (24.2%).

In describing the sample included in this study, 13.2% of children in RR and 4.9% in IO were younger than 6 months of age; 38.2% and 31.0%, respectively, were between 6 and 11 months old; and 48.7% and 64.1%, respectively, were between 12 and 23 months of age. Sixty percent of children included in the sample had all four determinations for micronutrients, including Hb, serum ferritin, serum zinc, and folic acid. The most common reason why the other 40% did not have complete lab determinations was because of insufficient blood sample. Only 3% of the samples were lipemic or hemolyzed, and thus unsuitable for analysis. Because of budgetary constraints, only half the samples were analyzed in the North and Center regions of IO; samples selected for analysis were drawn at random.

Results in Tables 2, 3, 4, 5, 6, 7 are shown by region and age group in each branch of IMSS. Resorting to the age groupings used in other published health surveys in the country, age categories included: less than 6 months, 6–11 months, and 12–23 months.

**Table 2:** Hemoglobin concentration and prevalence of anemia, by region, age group, and care regimen

**Region/Age groups**	**Regular Regimen (RR)**						**IMSS-Oportunidades (IO)**					
		
	*n*	*N*	Hemoglobin concentration g/dL*		Prevalence of anemia**		*n*	*N*	Hemoglobin concentration g/dL*		Prevalence of anemia**	
			Mean	95% CI	%	95% CI			Mean	95% CI	%	95% CI
		
**North**												
< 6 months	97	49416	11.5	10.9, 12.0	5.1	2.7, 9.6	142	992	11.7	11.3, 12.1	12.2	7.4, 19.4
6–11 months	321	181422	11.5	11.1, 11.9	14.5	9.0, 22.6	223	1891	11.6	11.2, 12.1	18.0	7.1, 38.4
12–23 months	367	223234	11.7	11.5, 11.9	15.2	10.6, 21.4	519	5073	11.7	11.3, 12.0	21.3	8.0, 45.9
**Total North**	**785**	**454072**	**11.6**	**11.2, 12.0**	**13.8**^a^	**9.8, 19.2**	**884**	**7956**	**11.7**	**11.3, 12.0**	**19.4**^a^	**8.2, 39.2**
												
**Center**												
< 6 months	148	53494	11.4	10.9, 11.8	4.5	1.4, 14.0	79	2315	13.7	10.9, 16.4	11.2	5.8, 20.4
6–11 months	291	115119	11.7	11.4, 12.0	14.4	9.7, 20.8	273	6975	12.1	11.4, 12.8	17.8	6.3, 41.1
12–23 months	324	151064	11.7	11.1, 12.2	19.1	13.9, 25.7	612	16858	12.2	11.6, 12.9	18.3	10.7, 29.5
**Total Center**	**763**	**319677**	**11.7**	**11.2, 12.2**	**15.0**^a^	**10.0, 21.8**	**964**	**26148**	**12.3**	**11.8, 12.9**	**17.6**^a^	**9.4, 30.4**
												
**South**												
< 6 months	27	14792	10.7	10.2, 11.2	31.3	20.6, 44.5	36	8701	11.3	10.9, 11.8	16.1	4.7, 42.5
6–11 months	227	78181	11.0	10.5, 11.4	36.1	20.0, 56.1	135	38100	11.6	11.4, 11.9	14.9	10.6, 20.5
12–23 months	351	110062	11.1	10.6, 11.6	39.2	23.1, 58.1	290	77882	11.8	11.5, 12.0	22.5	17.4, 28.6
**Total South**	**605**	**203035**	**11.0**	**10.5, 11.5**	**37.4**^a^	**22.6, 55.0**	**461**	**124683**	**11.7**	**11.5, 11.9**	**19.7**^a^	**16.4, 23.6**
												
**Mexico City ^&^**												
< 6 months	67	32810	10.9	10.7, 11.0	15.6	13.2, 18.3						
6–11 months	183	111755	11.2	11.0, 11.5	27.3	21.0, 34.6						
12–23 months	245	140561	11.4	11.2, 11.6	25.5	22.2, 29.1						
**Total Mexico City ^&^**	**495**	**285126**	**11.3**	**11.0, 11.5**	**25.1**^a^	**21.2, 29.3**						
												
**NATIONAL**												
< 6 months	339	150512	11.2	11.0, 11.4	9.8	6.3, 14.8	257	12008	11.8	11.0, 12.6	14.8	5.6, 33.6
6–11 months	1022	486477	11.4	11.2, 11.6	20.9	17.0, 25.4	631	46966	11.7	11.4, 11.9	15.5	11.2, 21.0
12–23 months	1287	624921	11.5	11.4, 11.7	22.7	19.2, 26.6	1421	99813	11.8	11.6, 12.1	21.7	17.3, 27.0
**TOTAL NATIONAL**	**2648**	**1261910**	**11.4**	**11.3, 11.6**	**20.5**^b^	**17.3, 24.1**	**2309**	**158787**	**11.8**	**11.6, 12.0**	**19.3**^b^	**16.1, 23.0**

*N *Population size (expanded sample)

* Hemoglobin concentration adjusted by altitude.

** Hemoglobin (Hb) < 13.5 g/dL for children under 2 weeks of age, Hb< 12.5 g/dL for children between 2 and 3 weeks of age, Hb <10 g/dL for children between 1 and 2 months old, Hb< 9.0 for children between 2 and 3 months old Hb< 9.5 for children between 3 and 6 months old, Hb<10.5 g/dL for children between 6 and <12 months old and Hb<10.7 g/dL for children between 12 and 23 months old.

^&^ Mexico City and periurban area

^a ^Comparing between regions, RR = 0.001; IO = 0.908.

^b ^Comparing between regimens, national level, *p *= 0.635

**Table 3:** Ferritin concentration and prevalence of iron deficiency, by region, age group, and care regimen

**Region/Age groups**	**Regular Regimen (RR)**						**IMSS-Oportunidades (IO)**					
		
	*n*	*N*	Ferritin concentration μg/L		Prevalence of iron deficiency*		*n*	*N*	Ferritin concentration μg/L		Prevalence of iron deficiency*	
			Mean	95% CI	%	95% CI			Mean	95% CI	%	95% CI
		
**North**												
< 6 months	84	40776	66.2	43.3, 89.0	4.3	2.6, 7.1	72	460	132.3	64.1, 200.5	6.1	2.3, 15.2
6–11 months	268	138802	30.6	25.2, 35.0	25.6	18.6, 34.2	139	1148	26.4	16.6, 36.2	36.9	30.1, 44.2
12–23 months	295	163476	16.1	12.8, 19.4	44.1	26.5, 63.3	332	3239	28.0	15.2, 40.8	43.3	32.6, 54.6
**Total North**	**647**	**343054**	**27.9**	**15.3, 40.5**	**31.9**^a^	**23.5, 41.6**	**543**	**4848**	**37.5**	**23.7, 51.4**	**38.2**^a^	**31.7, 45.2**
												
**Center**												
< 6 months	147	55228	96.4	56.2, 136.6	4.6	1.5, 13.0	33	703	65.6	8.5, 122.7	13.8	8.1, 22.4
6–11 months	282	119726	30.1	25.2, 35.0	19.4	16.2, 23.0	138	3644	21.1	4.6, 37.5	49.7	30.4, 69.1
12–23 months	348	177310	18.2	13.4, 23.0	39.0	33.4, 44.8	322	9268	25.8	1.0, 58.3	48.1	37.5, 58.8
**Total Center**	**777**	**352264**	**34.5**	**22.0, 47.0**	**26.8**^a^	**22.7, 31.4**	**493**	**13615**	**26.6**	**1.0, 54.1**	**46.7**^a^	**32.9, 61.1**
												
**South**												
< 6 months	25	14352	81.9	65.8, 97.9	21.0	13.5, 31.3	19	5007	50.4	33.1, 67.8	21.8	11.5, 37.5
6–11 months	239	79886	19.4	15.4, 23.4	37.3	30.4, 44.8	97	25128	59.3	24.0, 94.6	24.4	9.7, 49.1
12–23 months	401	123384	13.2	8.8, 17.5	54.4	43.8, 64.6	207	54992	35.8	16.7, 54.9	24.3	16.9, 33.6
**Total South**	**665**	**217622**	**20.2**	**12.0, 27.9**	**46.2**^a^	**37.4, 55.3**	**323**	**85127**	**43.6**	**18.6, 68.6**	**24.2**^a^	**16.0, 34.7**
												
**Mexico City ^&^**												
< 6 months	64	30872	87.9	70.5, 105.2	7.2	4.6, 11.2						
6–11 months	152	77977	32.2	28.9, 35.6	28.8	17.7, 43.4						
12–23 months	195	88543	26.4	16.2, 36.5	36.7	29.5, 44.4						
**Total Mexico City ^&^**	**411**	**197392**	**38.3**	**25.8, 50.8**	**28.9**^a^	**19.1, 41.1**						
												
**NATIONAL**												
< 6 months	320	141228	84.3	63.2, 105.5	6.8	4.3, 10.9	124	6170	58.3	41.2, 75.4	19.7	11.4, 31.8
6–11 months	941	416391	28.6	23.9, 33.3	26.7	22.8, 30.9	374	29920	53.4	21.7, 85.1	28.0	13.2, 49.7
12–23 months	1239	552713	17.8	15.1, 20.4	43.6	37.1, 50.2	861	67500	34.0	17.9, 50.2	28.4	22.3, 35.5
**TOTAL NATIONAL**	**2500**	**1110332**	**30.3**	**24.2, 36.4**	**32.6**^b^	**28.8, 36.6**	**1359**	**100230**	**41.1**	**20.0, 62.1**	**27.8**^b^	**20.2, 36.9**

*N *Population size (expanded sample)

* Ferritin <10 μg/L

^&^ Mexico City and periurban area

^a ^Comparing between regions within each regimen, RR = 0.015; IO = 0.007.

^b ^Comparing between regimens, national level, *p *= 0.287

**Table 4:** Iron nutritional status by hemoglobin and ferritin concentrations, by region and care regimen

	**Regular Regimen (RR)**																			
	**Total Regular Regimen** ***n *= 2324** ***N *= 1022247**				**North** ***n *= 639** ***N *= 337,221**				**Center** ***n *= 712** ***N *= 301,192**				**South** ***n *= 574** ***N *= 193,493**				**Mexico City and periurban area** ***n *= 399** ***N *= 190,340**			
	
**Iron status**	***n***	***N***	**%**	***95% CI ***	***n***	***N***	**%**	***95% CI ***	***n***	***N***	**%**	***95% CI ***	***n***	***N***	**%**	***95% CI ***	***n***	***N***	**%**	***95% CI ***
	
Normal ^a^	1298	582313	57.0^f^	52.1, 61.7	375	206383	61.2^g^	50.3, 71.1	499	203988	67.7^g^	58.9, 75.4	207	67839	35.1^g^	32.4, 37.8	217	104103	54.7^g^	41.9, 66.9
Iron deficiency without anemia ^b^	530	224550	22.0	18.2, 26.3	168	82056	24.3	17.8, 32.3	116	51229	17.0	14.6, 19.8	169	53701	27.8	16.0, 43.6	77	37563	19.7	14.9, 25.7
Anemia^c^	496	215385	21.0	17.1, 25.6	96	48781	14.5	11.0, 18.8	97	45976	15.3	10.1, 22.5	198	71953	37.2	22.7, 54.5	105	48675	25.6	18.6, 34.0
*Iron deficiency anemia *^d^	*248*	*105942*	*10.3*	*8.6, 12.4*	*49*	*24073*	*7.1*	*4.6, 10.9*	*59*	*30173*	*10.0*	*7.0, 14.1*	*102*	*34511*	*17.8*	*12.2, 25.4*	*38*	*17185*	*9.0*	*5.2, 15.3*
*Anemia due to other causes *^e^	*248*	*109443*	*10.7*	*8.0, 14.2*	*47*	*24708*	*7.3*	*4.8, 11.1*	*38*	*15803*	*5.2*	*2.5, 10.5*	*96*	*37442*	*19.4*	*10.2, 33.7*	*67*	*31490*	*16.5*	*13.0, 20.8*

**IMSS-Oportunidades (IO)**																				

	**Total IMSS-Oportunidades** ***n *= 1325** ***N *= 98150**				**North** ***n *= 539** ***N *= 4195**				**Center** ***n *= 479** ***N *= 10882**				**South** ***n *= 307** ***N *= 83074**							
	
**Iron status**	***n***	***N***	**%**	***95% CI ***	***n***	***N***	**%**	***95% CI ***	***n***	***N***	**%**	***95% CI ***	***n***	***N***	**%**	***95% CI ***				
	
Normal ^a^	681	60873	62.0^f^	53.2, 70.2	294	2308	55.0^h^	46.8, 63.0	192	4668	42.9^h^	33.9, 52.4	195	53897	64.9^h^	54.6, 73.9				
Iron deficiency without anemia ^b^	362	20110	20.5	13.8, 29.2	132	1140	27.2	21.0, 34.4	171	4240	39.0	29.3, 49.6	59	14730	17.7	10.9, 27.6				
Anemia^c^	282	17167	17.5	13.2, 22.8	113	746	17.8	9.1, 31.9	116	1974	18.1	8.0, 36.2	53	14447	17.4	12.7, 23.4				
*Iron deficiency anemia *^d^	*137*	*6873*	*7.0*	*4.7, 10.3*	*62*	*441*	*10.5*	*4.8, 21.4*	*57*	*984*	*9.0*	*4.2, 18.3*	*18*	*5448*	*6.6*	*4.0, 10.5*				
*Anemia due to other causes *^e^	*145*	*10294*	*10.5*	*7.4, 14.7*	*51*	*305*	*7.3*	*3.9, 13.2*	*59*	*990*	*9.1*	*3.6, 21.1*	*35*	*5448*	*10.8*	*7.4, 15.7*				

*N *Population size (expanded sample)

^a ^Hemoglobin above the cut-off point for anemia and ferritin > = 10 μg/L

^b ^Ferritin < 10 μg/L and Hb > = cut-off for anemia

^c ^Hb < cut-off for anemia

^d ^Ferritin < 10 μg/L and Hb < cut-off for anemia

^e ^Ferritin = 10 μg/L and Hb < cut-off for anemia

^f ^Comparing nutritional iron status between regimens *p *= 0.499

^g,h ^Comparing iron status between regions ^g^RR<0.001; ^h^IO = 0.012

**Table 5:** Folic acid concentration and prevalence of folic acid deficiency, by region, age group, and care regimen

**Region/Age groups**	**Regular Regimen (RR)**						**IMSS-Oportunidades (IO)**					
		
	*n*	*N*	Serum folic acid concentration ng/mL		Prevalence of folic acid deficiency*		*n*	*N*	Serum folic acid concentration ng/mL		Prevalence of folic acid deficiency*	
			Mean	95% CI	%	95% CI			Mean	95% CI	%	95% CI
		
**North**												
< 6 months	96	48932	102.7	51.0, 154.3	25.2	11.0, 47.9	97	601	124.8	105.0, 144.6	6.6	2.5, 16.3
6–11 months	298	176084	120.9	65.4, 176.3	14.5	3.2, 47.1	154	1169	120.5	98.6, 142.4	6.9	2.4, 18.3
12–23 months	314	196452	112.1	72.0, 152.2	13.4	2.7, 46.5	362	2640	125.8	110.8, 140.9	3.1	1.4, 6.5
**Total North**	**708**	**421468**	**114.7**	**55.9, 173.4**	**15.2**^a^	**3.3, 48.7**	**613**	**4410**	**124.3**	**109.2, 139.4**	**4.6**^a^	**2.3, 9.0**
												
**Center**												
< 6 months	142	49708	131.4	100.6, 162.2	9.7	2.2, 33.5	72	2065	163.6	149.4, 177.8	0.0	---
6–11 months	291	119236	149.0	122.1, 175.9	4.3	1.4, 13.0	239	6278	168.1	142.3, 193.9	3.0	0.8, 11.0
12–23 months	333	159516	127.5	99.6, 155.4	6.4	1.7, 21.7	546	15902	147.1	113.6, 180.7	1.4	0.2, 7.3
**Total Center**	**766**	**328460**	**135.9**	**103.8, 167.9**	**6.1**^a^	**1.7, 20.2**	**857**	**24246**	**154.0**	**124.9, 183.0**	**1.7**^a^	**0.4, 6.2**
												
**South**												
< 6 months	10	3973	118.7	58.3, 179.2	16.6	3.6, 51.7	25	6861	155.9	135.1, 176.7	0.0	---
6–11 months	202	62292	111.7	81.0, 142.4	8.6	1.6, 36.1	110	34142	134.4	108.5,160.3	5.8	2.4, 13.3
12–23 months	320	85651	111.4	88.4, 134.3	14.1	4.5, 36.1	228	68987	112.0	81.9, 142.1	12.6	3.0, 40.4
**Total South**	**532**	**151917**	**111.7**	**82.8, 140.5**	**11.9**^a^	**3.4, 34.2**	**363**	**109991**	**121.7**	**92.9, 150.5**	**9.7**^a^	**2.5, 30.6**
												
**Mexico City**^**&**^												
< 6 months	45	23968	107.0	103.6, 110.3	5.4	4.1, 7.2						
6–11 months	118	63812	121.3	108.7, 133.9	5.0	2.2, 11.0						
12–23 months	137	70367	106.8	104.2, 109.5	5.6	0.7, 32.1						
**Total Mexico City ^&^**	**300**	**158146**	**112.7**	**105.0, 120.3**	**5.1**^a^	**2.6, 10.1**						
												
**NATIONAL**												
< 6 months	293	126581	115.3	87.3, 143.2	15.1	5.8, 33.7	194	9528	155.6	139.9, 171.3	0.4	0.1, 1.4
6–11 months	909	421424	127.5	101.9, 153.2	9.3	3.2, 24.2	503	41589	139.1	119.7, 158.5	5.4	2.6, 11.0
12–23 months	1104	511986	116.0	98.0, 134.1	10.3	4.3, 22.5	1136	87530	118.8	95.2, 142.4	10.2	2.7, 32.0
**TOTAL NATIONAL**	**2306**	**1059991**	**120.5**	**99.3, 141.7**	**10.4**^b^	**4.0, 24.4**	**1833**	**138646**	**127.4**	**105.5, 149.4**	**8.1**^b^	**2.4, 23.7**

*N *Population size (expanded sample)

* ≤ 57 ng/mL in whole blood sample

^&^ Mexico City and periurban area

^a ^Comparing between regions within each regimen, RR = 0.458; IO = 0.046.

^b ^Comparing between regimens, national level, *p *= 0.720

**Table 6:** Zinc concentration and prevalence of low zinc concentration, by region, age group, and care regimen

**Region/Age Groups**	**Regular Regimen (RR)**						**IMSS-Oportunidades (IO)**					
		
	*n*	*N*	Zinc concentration μ/dL		Prevalence of low zinc concentration*		*n*	*N*	Zinc concentration μ/dL		Prevalence of low zinc concentration*	
			Mean	95% CI	%	95% CI			Mean	95% CI	%	95% CI
		
**North**												
< 6 months 6–11 months 12–23 months	94 289 330	46028 151419 189064	82.8 86.4 81.6	76.6, 89.0 74.0, 98.8 66.3, 96.9	20.6 22.7 31.2	8.1, 43.3 10.2, 43.2 14.6, 54.5	60 126 307	406 1104 2914	83.1 84.0 83.0	61.3, 104.9 70.7, 97.3 78.5, 87.6	35.2 21.6 21.1	11.0, 70.5 6.3, 53.1 8.8, 42.7
**Total North**	**713**	386511	**83.6**	**70.8, 96.4**	**26.6**^a^	**11.6, 50.1**	**493**	**4425**	**83.3**	**75.5, 91.0**	**22.5**^a^	**8.2, 48.7**
												
**Center**												
< 6 months 6–11 months 12–23 months	148 293 351	55798 123937 178793	76.3 76.1 75.1	70.5, 82.1 68.9, 83.2 73.3, 76.9	34.6 31.3 32.3	20.3, 52.2 23.1, 41.0 25.6, 39.8	33 131 315	981 3516 9230	72.5 79.8 79.7	49.9, 95.1 73.2, 86.5 74.3, 85.1	63.2 29.4 26.6	30.1, 87.2 21.4, 38.8 19.2, 35.6
**Total Center**	**792**	**339398**	**75.6**	**71.7, 79.6**	**32.4**^a^	**26.0, 39.4**	**479**	**13727**	**79.2**	**75.4, 83.0**	**29.9**^a^	**24.1, 36.5**
												
**South**												
< 6 months 6–11 months 12–23 months	29 247 404	15312 81878 124130	72.4 77.7 78.6	64.8, 79.9 72.7, 82.7 71.6, 85.7	38.1 25.6 26.1	21.2, 58.5 17.3, 36.2 18.4, 35.6	25 100 211	6605 25968 55166	87.5 91.6 90.4	74.9, 100.2 84.7, 98.5 82.9, 97.9	5.9 14.4 9.0	1.4, 22.1 6.6, 28.8 3.2, 22.8
**Total South**	**680**	**222491**	**77.9**	**71.8, 83.9**	**26.6**^a^	**18.4, 36.8**	**336**	**87740**	**90.5**	**84.3, 96.8**	**10.4**^a^	**5.2, 19.5**
												
**Mexico City ^&^**												
< 6 months 6–11 months 12–23 months	60 161 198	28968 82599 89905	87.5 81.6 88.2	70.4, 104.5 65.2, 98.0 73.0, 103.3	20.2 25.6 22.5	4.0, 60.6 19.0, 33.6 11.8, 38.8						
**Total Mexico City ^&^**	**419**	**211391**	**85.4**	**69.1, 101.7**	**24.0**^a^	**12.8, 40.4**						
												
**NATIONAL**												
< 6 months 6–11 months 12–23 months	331 990 1283	146106 439833 581893	80.1 81.0 80.0	74.3, 85.9 74.9, 87.1 74.4, 85.6	27.7 26.2 29.1	16.6, 42.4 19.6, 34.2 22.2, 37.1	118 357 833	7992 30588 67311	805 90.0 88.6	74.6, 96.3 83.7, 96.2 82.4, 94.9	14.4 16.4 12.0	6.7, 28.3 9.0, 27.8 6.2, 21.7
**TOTAL NATIONAL**	**2604**	**1179791**	**80.4**	**74.9, 85.8**	**27.9**^b^	**20.9, 36.1**	**1332**	**108912**	**88.8**	**83.6, 94.0**	**13.4**^b^	**8.3, 21.0**

*N *Population size (expanded sample)

* Zinc concentration < 65 μ/dL

^&^ Mexico City and periurban area

^a ^Comparing between regions within each regimen, RR = 0.746; IO = 0.003.

^b ^Comparing between regimens, national level, *p *= 0.005

**Table 7:** Folic acid deficiency and low zinc concentration by iron status

	**Regular Regimen (RR)**							
		
**Iron status**	**Folic acid deficiency by iron status ^h^** ***n *= 2025** ***N *= 856,699**				**Low zinc concentration by iron status^i^** ***n *= 2268** ***N *= 996,996**			
		
	**Cases/*n***	***N***	**%**^f^	**95% CI**	**Cases/*n***	***N***	**%**^g^	**95% CI**
Normal^a^	91/1135	493903	8.3	2.5, 24.1	328/1264	567943	26.1	19.4, 34.1
Iron deficiency without anemia^b^	43/474	194059	10.8	3.8, 26.9	130/515	216433	25.8	18.4, 34.9
Anemia^c^	80/416	168736	19.3	10.5, 32.8	139/489	212621	30.2	22.1, 39.3
*Iron deficiency anemia*^d^	*39/214*	*84405*	*18.5*	*10.9, 29.7*	*76/246*	*105359*	*32.7*	*23.0, 44.3*
*Anemia due to other causes *^e^	*41/202*	*84331*	*20.1*	*8.9, 39.5*	*63/243*	*107262*	*27.8*	*18.3, 39.8*

**IMSS-Oportunidades (IO)**								
	
**Iron status**	**Folic acid deficiency by iron status^h^** ***n *= 1062** ***N *= 88,848**				**Low zinc concentration by iron status ^i^** ***n *= 1214** ***N *= 92,310**			
		
	**Cases/*n***	***N***	**%**^f^	**95% CI**	**Cases/*n***	***N***	**%**^g^	**95% CI**

Normal^a^	27/517	55564	8.8	2.6, 25.5	128/616	56805	11.4	5.3, 22.8
Iron deficiency without anemia^b^	8/298	18733	8.3	1.5, 34.5	80/334	19425	9.9	6.2, 15.4
Anemia^c^	18/247	14550	12.8	2.4, 46.8	85/264	16080	15.1	7.9, 26.8
*Iron deficiency anemia*^d^	*11/122*	*6143*	*23.8*	*6.0, 60.5*	*39/128*	*6753*	*8.2*	*3.8, 16.7*
*Anemia due to other causes*^e^	*7/125*	*8407*	*4.8*	*0.3, 45.8*	*46/136*	*9327*	*20.1*	*8.8, 39.5*

*N *Population size (expanded sample)

^a ^Hemoglobin above the cut-off point for anemia and ferritin > = 10 μg/L

^b ^Ferritin < 10 μg/L and Hb > = cut-off for anemia

^c ^Hb < cut-off for anemia

^d ^Ferritin < 10 μg/L and Hb < cut-off for anemia

^e ^Ferritin ≥ 10 μg/L and Hb < cut-off for anemia

^f ^Comparing folic acid deficiency between iron status, RR *p *= 0.102; IO *p *= 0.208

^g ^Comparing low zinc concentration between iron status, RR *p *= 0.444; IO *p *= 0.238

^h ^Comparing folic acid deficiency between RR and IO according to iron status (*p *= 0.387)

^i ^Comparing low zinc concentration between RR and IO according to iron status (*p *= 0.220)

### Prevalence of anemia

In RR, the prevalence of anemia for the whole country was 20.5%, with significant differences between regions. The highest prevalence of anemia was found in the South (37.4%) and in Mexico City and its periurban area (25.1%) (Table 2) (*p *= 0.001). Prevalence of anemia at the national level was highest in children between 12 and 23 months of age (22.7%) and lowest in children under 6 months of age (9.8%); children between 6 and 11 months old showed 20.9% prevalence. In IO, prevalence of anemia at national level was 19.3%, with no statistically significant differences in prevalence between regions (Table 2). There were no statistically significant differences in the prevalence of anemia between RR and IO (*p *= 0.635).

### Prevalence of iron deficiency

The prevalence of iron deficiency, determined by ferritin concentration <10 μg/L, in RR was 32.6% (Table 3). Body iron stores were more depleted as age increased: in children younger than 6 months of age the prevalence was 6.8%; for children between 6 and 11 months, it was 26.7%; and for children 12–23 months old, it was 43.6%. There were marked differences in overall prevalence between regions, with the lower and higher values in the Center (26.8%) and South (46.2%), respectively (*p *= 0.015) (Table 3).

In IO, iron deficiency had a prevalence of 27.8%. As described for RR, iron deficiency increased with age: in children younger than 6 months of age, prevalence of iron deficiency was 19.7%; in children between 6 and 11 months old, prevalence was 28.0%; and in children 12–23 months old, prevalence of iron deficiency was 28.4%. The highest prevalence of iron deficiency was found in the Center (46.7%), followed by the North (38.2%) and by the South (24.2%) (*p *= 0.007). There were no statistically significant differences in the overall prevalence of iron deficiency between regimens (*p *= 0.287).

Other studies have used a cut-off for ferritin concentration <12 μg/L to evaluate low iron stores in this age group, so we ran a second analysis with this cut-off. In RR, the prevalence of iron deficiency at the national level was 37.0% (95% CI: 33.6, 40.6), and by regions, the North showed 36.3% (95% CI: 28.1, 45.4), the Center, 32.4% (95% CI: 28.4, 36.7), the South, 50.8% (95% CI: 42.5, 59.2), and Mexico City and periurban area, 31.5% (95% CI: 23.0, 41.4). In IO, the national prevalence was 32.1% (95% CI: 23.7, 41.9); in the North, 42.4% (95% CI: 37.5, 47.4); in the Center, 50.2% (95% CI: 35.5, 64.9); and in the South, 28.7% (95% CI: 19.6, 39.9). Between regions there were statistically significant differences, with a *p *= 0.006 for RR and *p *= 0.017 for IO, but the prevalence of iron deficiency between the two IMSS regimens was not statistically significantly different (*p *= 0.326).

### Iron status by hemoglobin and ferritin concentration

In RR, 57.0% of all children had a normal iron status, as judged by normal hemoglobin and ferritin concentrations. Thus, 43% of children had iron deficiency and/or anemia. Only half (10.3%) of the 21% of cases with anemia were associated with iron deficiency. The highest prevalence of both iron deficiency without anemia and anemia was found among children in the South, where only 35.1% of children had normal iron status; this was followed by Mexico City and its periurban area, with 54.7%. In the North, 61.2% of children had normal iron status; in the Center, 67.7% did (*p *< 0.001) (Table 4).

In IO, 62.0% of children had normal iron status. The rest (38.0%) had iron deficiency and/or anemia. Of all cases of anemia found in IO (17.5%), fewer than half (7.0%) were due to iron deficiency; the rest (10.5%) were from other causes. In the Center, only 42.9% of children had normal iron status, compared to 55.0% of children in the North and 64.9% of children in the South (*p *= 0.012) (Table 4). There were no statistically significant differences in the overall prevalence of iron deficiency without anemia and anemia between regimens (*p *= 0.499).

### Iron status according to hemoglobin and ferritin concentration, by age

**Figure 1:**
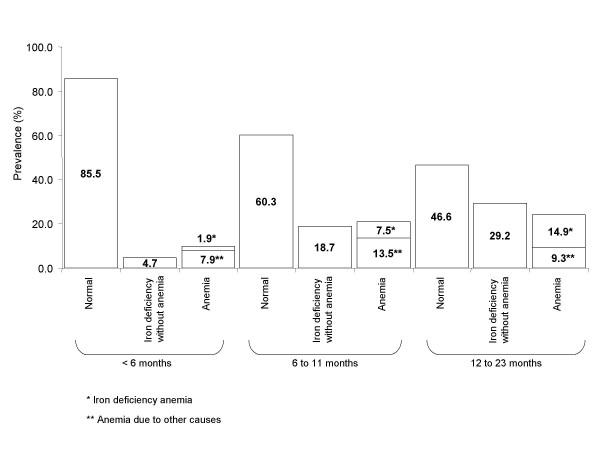
**Iron nutritional status according to hemoglobin and ferritin values, by age group, in the regular regimen (RR)**. Normal: Hemoglobin above the cut-off for anemia and ferritin > = 10 μg/L. Iron deficiency without anemia: ferritin < 10 μg/L and Hb > = cut-off for anemia, by age group. Anemia: Hb < cut-off for anemia, by age group. Iron deficiency anemia: ferritin < 10 μg/L and Hb < cut-off for anemia, by age group. Anemia due to other causes: ferritin ≥ 10 μg/L and Hb < cut-off for anemia, by age group.

Figure 1 shows data on iron status by age for the whole RR regimen. The largest percentage of normal iron status was found among children younger than 6 months of age. However, in the South, only 64.9% of children had normal iron status at this age; in Mexico City and its periurban area, 76.6% did; and in the North and Center, more than 90% did. We found 9.8% prevalence of anemia in this age group. It is worth noting that between 6 and 11 months of age, 18.7% of children had iron deficiency without anemia and 21% had anemia. Throughout the first year of life, there was a greater prevalence of anemia not associated with iron deficiency as compared with iron-deficiency anemia (7.9 vs. 1.9% and 13.5% vs. 7.5% in children less than 6 months of age and between 6 and 11 months of age, respectively).

Among children in their second year of life, only 46.6% had normal iron status, with the lowest percentage in the South (28.4%). Iron deficiency without anemia in this age group had a prevalence of 29.2%, with an increasing prevalence of iron-deficiency anemia (14.9%), as compared with 9.3% of anemia due to other causes.

**Figure 2:**
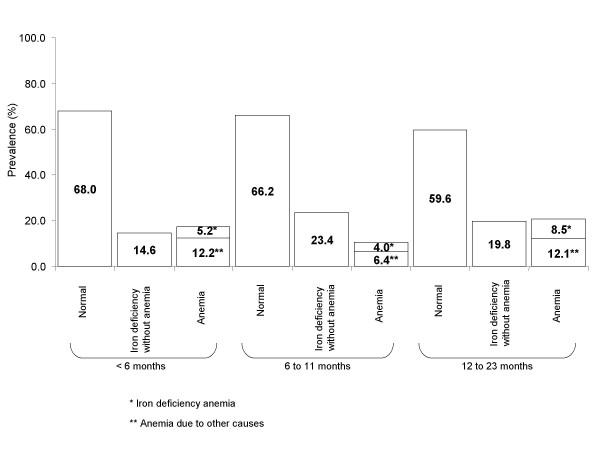
**Iron nutritional status according to hemoglobin and ferritin values, by age group, in IMSS-Oportunidades (IO)**. Normal: Hemoglobin above the cut-off for anemia and ferritin > = 10 μg/L. Iron deficiency without anemia: ferritin < 10 μg/L and Hb > = cut-off for anemia, by age group. Anemia: Hb < cut-off for anemia, by age group. Iron deficiency anemia: ferritin < 10 μg/L and Hb < cut-off for anemia, by age group. Anemia due to other causes: ferritin ≥ 10 μg/L and Hb < cut-off for anemia, by age group.

Figure 2 shows data on iron status by age for the whole IO regimen. The largest percentage of children with normal iron nutritional status was found among those younger than 6 months of age. However, in the South and Center, only 66.4% and 69.1% of children, respectively, had normal iron status at this age, compared to the North, where 84.6% of children had normal iron status. Prevalence of iron deficiency without anemia even at this early age was 14.6%, with the highest prevalence (15.9%) in the South. During the 6 first months of life, prevalence of anemia was 17.4%; however, iron deficiency was associated with anemia only in 5.2% of cases. In children 6–11 months old, prevalence of iron deficiency without anemia was 23.4%; the prevalence nearly doubled (40.4%) in the Center. Anemia prevalence was 10.4%, with the highest prevalence (18.1%) in the Center. In IO, only 59.6% of children between 12 and 23 months of age had normal iron status. The lowest prevalence of children with normal iron status (41.3%) was found in the Center. Prevalence of iron-deficiency anemia corresponded to 8.5%; 12.1% corresponded to anemia due to other causes (i.e., associated with normal ferritin values).

### Prevalence of folic acid deficiency

There was a 10.4% prevalence of folic acid deficiency in RR at a national level; it was highest in the North (15.2%) and lowest in Mexico City (5.1%) (Table 5). By age group, children under 6 months old had the highest prevalence (15.1% at national level). In contrast, children living in IO had an 8.1% prevalence of folic acid deficiency, with highest in the South (9.7%) and lowest in the Center (1.7%) (Table 5). Also in contrast to the findings in RR, prevalence of folic acid deficiency was lowest in children under 6 months of age (0.4%); folic acid deficiency in this age group was only found in the North. By age group, the highest prevalence was found in children between 12 and 23 months (10.2%). When comparing between regimens, the difference in the prevalence of folic acid deficiency was not statistically significant (*p *= 0.72).

### Prevalence of low zinc concentrations

Prevalence of low serum zinc concentrations was 27.9% in RR and 13.4% in IO (*p *= 0.005). The Center had the highest prevalence of low zinc concentration, both in RR (32.4%) and in IO (29.9%). Although there were no statistically significant differences in the prevalence of low zinc concentrations by region in RR, these were statistically significantly different in IO, the highest prevalence found in the Center (29.9%) and the lowest in the South (10.4%). (Table 6).

### Multiple micronutrient deficiencies: folic acid and zinc according to iron nutritional status

Prevalence of folic acid deficiency according to iron nutritional status was not statistically significant different when comparing RR with IO. In RR, children with normal iron status had a prevalence of folic acid deficiency of 8.3%; children with iron deficiency without anemia had a prevalence of folic acid deficiency of 10.8%; and those with anemia had 19.3%: folic acid deficiency were found in 18.5% of children with anemia because of iron deficiency, and 20.1% was found in children with anemia due to other causes (*p *= 0.10). Similarly, in IO the prevalence of folic acid deficiency was 8.8% in children with normal iron status, 8.3% in iron-deficient children without anemia, and 12.8% in children with anemia: 23.8% in children with anemia due to iron deficiency and 4.8% with anemia due to other causes (*p *= 0.21), (Table 7).

Prevalence of low zinc concentrations according to iron status was also not statistically significantly different when comparing RR and IO. Children in RR who showed normal iron status had a 26.1% prevalence of low zinc concentrations; children with iron deficiency without anemia had a prevalence of 25.8%; those with anemia had 30.2% prevalence, with 32.7% in iron-deficiency anemia and 27.8% in anemia due to other causes (*p *= 0.44). Children in IO with normal iron status had a prevalence of low zinc concentrations in 11.4% of cases; children with iron deficiency without anemia had low zinc concentration in 9.9%; children with anemia had 15.1%: 8.2% corresponded to children with iron-deficiency anemia and 20.1% in those with anemia due to other causes (*p *= 0.24) (Table 7).

In RR, 1.2% of children had iron, zinc, and folic acid deficiencies simultaneously; 3.0% had zinc and folic acid deficiencies; 4.3% had iron and folic acid deficiencies; and 9.2% had zinc and iron deficiencies. In IO, 0.2% of children had simultaneous iron, zinc, and folic acid deficiencies; 0.8% had zinc and folic acid deficiencies; 3.4% had iron and folic acid deficiencies; and 2.7% had zinc and iron deficiencies.

In RR, 8.2% of children with anemia had simultaneous zinc and folic acid deficiencies; 7.0% corresponded to children with iron-deficiency anemia; and 9.4% corresponded to children with anemia due to other causes.

In IO, 0.3% of children with anemia had simultaneous folic acid and zinc deficiencies: 0.6% of children had iron-deficiency anemia, and 0.1% had anemia from other causes. Folic acid and zinc deficiencies were present in 0.9% of children with iron deficiency without anemia and in 1.0% of children with normal iron status.

## Discussion

The evidence presented in this article is disturbing in terms of public nutrition. It shows that children younger than 2 years old have several micronutrient deficiencies that may stop them from attaining their full growth and development potentials. One of every five children had anemia, and one of every three had iron deficiency. More than half the anemia cases were not accompanied by low ferritin levels, reflecting that they may be from causes other than iron deficiency. Low serum zinc concentrations were found in one out of three children living in the urban areas and in one out of ten of those living in rural areas; 10% of children of RR and 8% of children in IO had folic acid deficiencies. Low serum zinc and iron deficiency appeared together in 9% and 3% of children living in urban and rural areas, respectively.

Although we have the largest sample size published in the country in this age group, there are several possible sources of bias in our sample. The sample was only representative of children who use the health care services provided by the institution and who were free of acute infectious diseases during the two weeks before giving the blood sample. Although the decision to exclude data about children with acute infectious diseases ensured that the values of iron and zinc would not be affected by the infectious state, it is possible that children who were ill may have had a different micronutrient status than those included in the sample, introducing a further potential bias.

In RR, our study sample was only 64.2% of the calculated sample size; in IO, it was 74.7%; with differences by regions. In view of the different response rates, which led to underrepresentation of the South and Mexico City regions for RR and the South region for IO, there is a likelihood of biases in some of the results presented. The different regions in the country have noticeable contrasts in socioeconomic indicators, as the North is more developed than the South. Likewise, the two regimens care for populations with noticeable differences in economic development, favoring the urban areas over the rural/semi-rural. Other possible sources of bias emerge from the composition of the subsample of children who participated in the micronutrient study. In comparison with the rest of the children who participated in the larger study, those who were sampled for micronutrient status were older. Also, children in the IO subsample had lower weight for age Z scores. Therefore, it is possible that the true prevalence might be overestimated, as older children and those with lower weight for age may also be more likely to have micronutrient deficiencies. We also can not rule out the possibility that some characteristics of the study sample might have been influenced by parental acceptance of the study, which might have been affected by their own perceptions and concerns about their children's health status. It is difficult to estimate how the different sampling biases might have influenced the results, however, we are confident that, whatever the direction of the biases, the sampled population reflects the population who use the primary health care facilities of IMSS. To correctly interpret our results, it is necessary to consider some methodological issues. The criterion that we used to define iron deficiency was based only on ferritin values, defining a cutoff of <10 μg/L to reflect low iron stores. Other indicators of iron status, like the percentage of transferrin saturation (with a cut-off at <10%) were not useful, as it only classified 0.2% and 3.3% of children in RR and IO, respectively, as iron deficient. This may be because transferrin saturation has ample variation in the first year of life, so there is no agreement on the best cut-off [16,30,31]. In view of the low sensitivity that this indicator has to detect iron deficiency in this age group, we decided not to use it.

There is no general agreement about the preferred cut-off point for ferritin in this age group. We used a cut-off of < 10 μg/L, but other authors suggest a different one. Some authors have pointed out that the cut-off values for children under 5 should vary between 8 and 12 μg/L, depending on the specific age group [32-36]. WHO [36] recommends using a cut-off of < 12 μg/L. We ran our analysis with this WHO-recommended cut-off and found a difference in prevalence of about 4 percentage points higher with it. In NHANES III (1988–1994), the value of the 5^th ^percentile for serum ferritin in children under 1 year of age was equivalent to 11 μg/L; the corresponding value for children between 1 and 2 years of age was 6 μg/L. However, for the Mexican American sample included in this study, the corresponding values were 8 and 3 μg/L, respectively [37]. Looking at healthy Honduran and Swedish children–exclusively breast-fed infants at 4 and 6 months of age–Domellöf et al. found -2 SD cut-off values corresponding at 4 mo of age to 20 μg/L, at 6 mo of age to 9 μg/L, and at 9 mo of age, to 5 μg/L [38]. Soh et al. have summarized this controversy, stating that until the validity of cut-off points of iron indices is confirmed, prevalence estimates of iron deficiency in children younger than 2 must remain conjectural [39].

Anemia, on the other hand, was defined based on hemoglobin values that were adjusted for each age category in the range of our study. The issue here is whether these cut-offs are adequate to identify anemia. The use of a single cut-off for children under 5 years of age has been a point for discussion [24]. Some authors consider that 11 g/dL, as proposed by WHO for this age group, may be too high for children less than 2 years old [16,30,34]. In fact, changes in iron metabolism found in this age, characterized by a rapid growth spurt and exposure to different infectious diseases, many of which happen subclinically, may affect the biochemical response of the iron status indicators, so there is uncertainty about the proper cut-offs that may best reflect functional outcomes [16,30]. Sheriff et al. found that the 5th percentile for hemoglobin values for toddlers 8 months of age was 9.7 g/dL, whereas the corresponding value for children between 12 and 18 months was 10 g/dL [34]. Further, a study carried out in Sweden with healthy babies born at term found that more than 30% had hemoglobin concentrations under 11 g/dL by 6 months of age, although fewer than 3% had ferritin values less than 12 μg/L, so few had low iron stores [16].

In our own study, more than 50% of cases with hemoglobin under the age-specific cut-off were not associated with low ferritin values. It may be that, even when we used cut-off values adjusted for age, we overestimated the prevalence of anemia. The possibility exists that, although we left out any child with data of acute infection and our sample population was mostly drawn from the well-baby clinic and preventive programs, a certain proportion of cases might have had a subclinical infection that raised ferritin values; however, we do not think that this is the most likely explanation. Therefore, children who presented low hemoglobin and normal ferritin values were considered as having anemia from causes other than iron deficiency.

Other micronutrient deficiencies are also known to cause anemia; this is the case of folic acid deficiency, as well as others, like vitamin A and B_12_, which were not assessed in our study [40]. Anemia without iron deficiency has been reported by other authors, in some instances in as many as over 50% of cases, a prevalence similar to the one found in our study [25,41,42]. It should be noted that this last figure also holds true when we analyzed our data using a cut-off of < 12 μg/L for serum ferritin to identify iron deficiency.

Even when most of the Mexican population is covered by the IMSS system, this population has several different characteristics. On the one hand, for RR, at least one of the parents has to have a regular salary to be an IMSS beneficiary. On the other hand, the populations served by IO are also beneficiaries of other subsidy and nutrition alleviation government programs, such as Oportunidades [43]. This and other programs are targeted at low-income households, and include the provision of micronutrient-fortified products for pregnant/lactating women and infants between the ages of 4 and 23 months old, as well as for underweight children aged 2–4 and may have some influence in improving the micronutrient status of these groups [44]. Thus, it is relevant to compare our data with those collected by the NNS conducted in 1999, in Mexico as both studies were carried out at approximately the same time. However, there were some methodological differences: our survey used venous blood for sampling, whereas NNS used capillary samples; our study used age-adjusted hemoglobin cut-offs values to identify anemia, whereas NNS used two cut-offs: 9.5 g/dL for children 6–11 months old (compared to 10.5 g/dL used in the present study for this age group), and 11 g/dL for children 12–23 months old (compared to 10.7 g/dL for this age group in our study) [13].

With these caveats in mind, we noted that children 6–11 months old in RR had a prevalence of anemia of 20.9 (95% CI = 17.0–25.4), higher than that found by NNS: 11.3% (95% CI = 11.2–21.0). In comparison, the prevalence found in children in IO was very similar to the one found in the NNS rural areas of the country: 15.5%, 95% CI = 11.2–21.0 vs. 16.2%, 95% CI = 11.8–20.6, respectively. In our study, children between 12 and 23 months old had lower prevalence of anemia than the general Mexican population reported by NNS, both in the RR and the IO areas: 22.7%, 95% CI = 19.2–26.6 vs. 46.8%, 95% CI = 43.1–50.5 in urban areas and 21.7%, 95% CI = 17.3–27.0 vs. 52.9%, 95% CI = 48.0–57.9, for the IMSS and the general population, respectively. Taking an external reference as comparison, in the 2002 report of the WHO for Latin America, the prevalence of anemia in children under 5 varied between 16% and 28% [45]. Although the prevalence that we found in our study lies well within this range, it is known that the prevalence of anemia is higher in the first 2 years of life. Therefore, the prevalence that we found in our population may be lower than that in other Latin American countries and was lower than that found in the general Mexican population.

Another interesting comparison was found when contrasting iron deficiency between RR and IO. In both regimens, iron deficiency without anemia and anemia were present since the first 6 months of life in 15% of children under RR and 32% of children under IO. Iron deficiency at this early age has often been found to be related to a combination of the poor iron stores accumulated over the last trimester of pregnancy and the poor quality of complementary foods, which may be either low in iron or in sources of highly bio-available iron [18,39]. Further, it was clear that the prevalence of iron deficiency increased with age, a fact that calls attention to the need for early actions by primary health care public services.

With respect to other micronutrient deficiencies, the only statistical significant difference between RR and IO was a higher prevalence of low serum zinc concentration in the former. Although it would be expected to find higher prevalences of micronutrient deficiencies in the more underprivileged areas, the targeted food and nutritional assistance that the underserved population has received over the past years may be reflected in an improvement in their micronutrient status [44]. It is generally accepted that we do not have good indicators to assess zinc nutritional status, especially as related to biological responses that may translate functional outcomes of mild to moderate deficiency. Some studies have evaluated the adequacy of dietary consumption, whereas others have used plasma or serum concentration of zinc. The WHO has estimated the prevalence of zinc deficiency based on dietary intake, which has a mean of 31% worldwide, ranging between 6 and 73% in different populations in the under-5-year-old group [45]. The most widely used indicator, however, has been plasma or serum zinc concentration [7,26]. Using this indicator, we found a high prevalence of zinc deficiency, particularly in children of RR. Even when the consequences of zinc deficiency have been fairly well documented in the literature and include limitations on growth potential, impaired psycho-motor development, impaired immune function, and delayed bone and sexual maturation onset [5-7,46,47], the extent of zinc deficiency has not been properly established worldwide, nor are there specific public nutrition programs addressing it, aside from the recent zinc supplementation recommended for children with acute diarrhea [5,46,48].

Another result worth discussing is the presence of multiple micronutrient deficiencies. When compared to children with normal iron status, children with anemia had a higher prevalence of folic acid deficiency. Similarly, low plasma zinc concentrations were more prevalent in children with anemia. Although the prevalence of children with three simultaneous deficiencies of the micronutrients studied was relatively low, the coincidence of two of these deficiencies singled out those of zinc and iron as the most common deficiencies in RR children, and iron and folic acid in IO. These deficiencies will manifest themselves as anemia–not necessarily related to iron deficiency. They call for more comprehensive interventions–whether food based, supplement based, or fortification based–than the ones usually considered by public health programs. Further, the early age at which these deficiencies present themselves is a call for public health interventions to consider preventive approaches rather than curative ones.

## Conclusion

Iron and zinc are the principal micronutrient deficiencies in Mexican children younger than 2 years old who use the health care services provided by IMSS. Anemia not associated with low ferritin values was more prevalent than iron-deficiency anemia. The fact that we found multiple micronutrient deficiencies at this early age reveals a need to establish effective preventive public health nutrition programs to address them.

## Competing interests

The author(s) declare that they have no competing interests.

## Authors' contributions

The authors' contributions to this publication are as follows: designing the study, analyzing the data, writing the manuscript, and obtaining funding for this research (MXDL, SFH, SFH, IMR, SM, HMS); supervising the project, evaluating data, reviewing the draft, and contributing comments for the final manuscript (MEM, BT, GMA, RIR, MGU). All authors read and approved the final manuscript.

## Pre-publication history

The pre-publication history for this paper can be accessed here:


